# CPT1A as a potential therapeutic target for lipopolysaccharide-induced acute lung injury in mice

**DOI:** 10.1038/s41598-024-52042-2

**Published:** 2024-01-18

**Authors:** Gui-Yun Wang, Xia Xu, Da-Yan Xiong, Lang Deng, Wei Liu, Xiao-Ting Huang

**Affiliations:** 1 Shandong Xiehe University, Jinan, 250109 Shandong China; 2 Xiangya School of Nursing, Central South University, Changsha, 410013 Hunan China

**Keywords:** Diseases, Medical research, Molecular medicine, Pathogenesis

## Abstract

Acute lung injury (ALI) remains a high mortality rate with dramatic lung inflammation and alveolar epithelial cell death. Although fatty acid β-oxidation (FAO) impairment has been implicated in the pathogenesis of ALI, whether Carnitine palmitoyltransferase 1A (CPT1A), the rate-limiting enzyme for FAO, plays roles in lipopolysaccharide (LPS)-induced ALI remains unclear. Accordingly, we focused on exploring the effect of CPT1A in the context of ALI and the underlying mechanisms. We found that overexpression of CPT1A (AAV-CPT1A) effectively alleviated lung injury by reduction of lung wet-to-dry ratio, inflammatory cell infiltration, and protein levels in the BALF of ALI mice. Meanwhile, AAV-CPT1A significantly lessened histopathological changes and several cytokines’ secretions. In contrast, blocking CPT1A with etomoxir augmented inflammatory responses and lung injury in ALI mice. Furthermore, we found that overexpression of CPT1A with lentivirus reduced the apoptosis rates of alveolar epithelial cells and the expression of apoptosis-related proteins induced by LPS in MLE12 cells, while etomoxir increased the apoptosis of MLE12 cells. Overexpression of CPT1A prevented the drop in bioenergetics, palmitate oxidation, and ATP levels. In conclusion, the results rendered CPT1A worthy of further development into a pharmaceutical drug for the treatment of ALI.

## Introduction

Acute lung injury (ALI), a severe clinical condition of acute respiratory distress syndrome (ARDS), has a high mortality rate and substantially impacts public health^1–3^. ALI is a disorder of acute inflammation following infection, trauma, or aspiration of gastric contents, characterized by exaggerated production of pro-inflammatory mediators, infiltration of inflammatory cells, and apoptosis of alveolar epithelial cells (AECs)^4–7^. Although numerous studies focus on the therapeutic strategy and understanding of the mechanism, the annual mortality rate of ALI remains high. Therefore, it is urgently required to explore novel therapeutic targets and methods for treating clinical ALI.

Alveolar type II epithelial cells (AEC II) are the essential part of lung epithelial cells, which could regulate alveolar surface tension in the lungs by secreting all surfactant components^8,9^. AEC II are the primary targeting cells for injuries^10,11^. Epithelial injuries occurring during the development of ALI may result from the apoptosis of AECs^12,13^. Apoptosis of AECs has been well recognized as one of ALI's main mechanisms^14,15^. Protecting AEC II cells from apoptosis could alleviate the ALI in mice^16–18^.

Carnitine palmitoyltransferase 1A (CPT1A) is the rate-limiting enzyme for the fatty acid β-oxidation (FAO)^19,20^. CPT1A mediates the β-oxidation of fatty acid by catalyzing the cellular long-chain ester acyl CoA of fatty acids into long-chain ester acylcarnitine. Then it transports fatty acids across the mitochondrial membrane into the matrix for oxidative energy supply^20,21^. CPT1A is mainly expressed in the liver, kidney, and lung^22–25^. It has been reported that FAO impairment is an important contributing factor to the pathogenesis of ALI^2^. CPT1A is remarkably reduced in lung tissue and AECs of ALI mice induced by lipopolysaccharide (LPS)^2^. However, the potential role of CPT1A in ALI and LPS-induced AECs dysfunction remains unclear.

In the present study, we explored the role of CPT1A in LPS-induced ALI. Furthermore, we investigated the effect of CPT1A on inflammation and cell apoptosis. These findings may shed new insights into the treatment of ALI (Supplementary information).

## Results

### CPT1A overexpression alleviated lung injury and suppressed the inflammatory response in lung tissue of ALI mice induced by LPS

**Figure 1:**
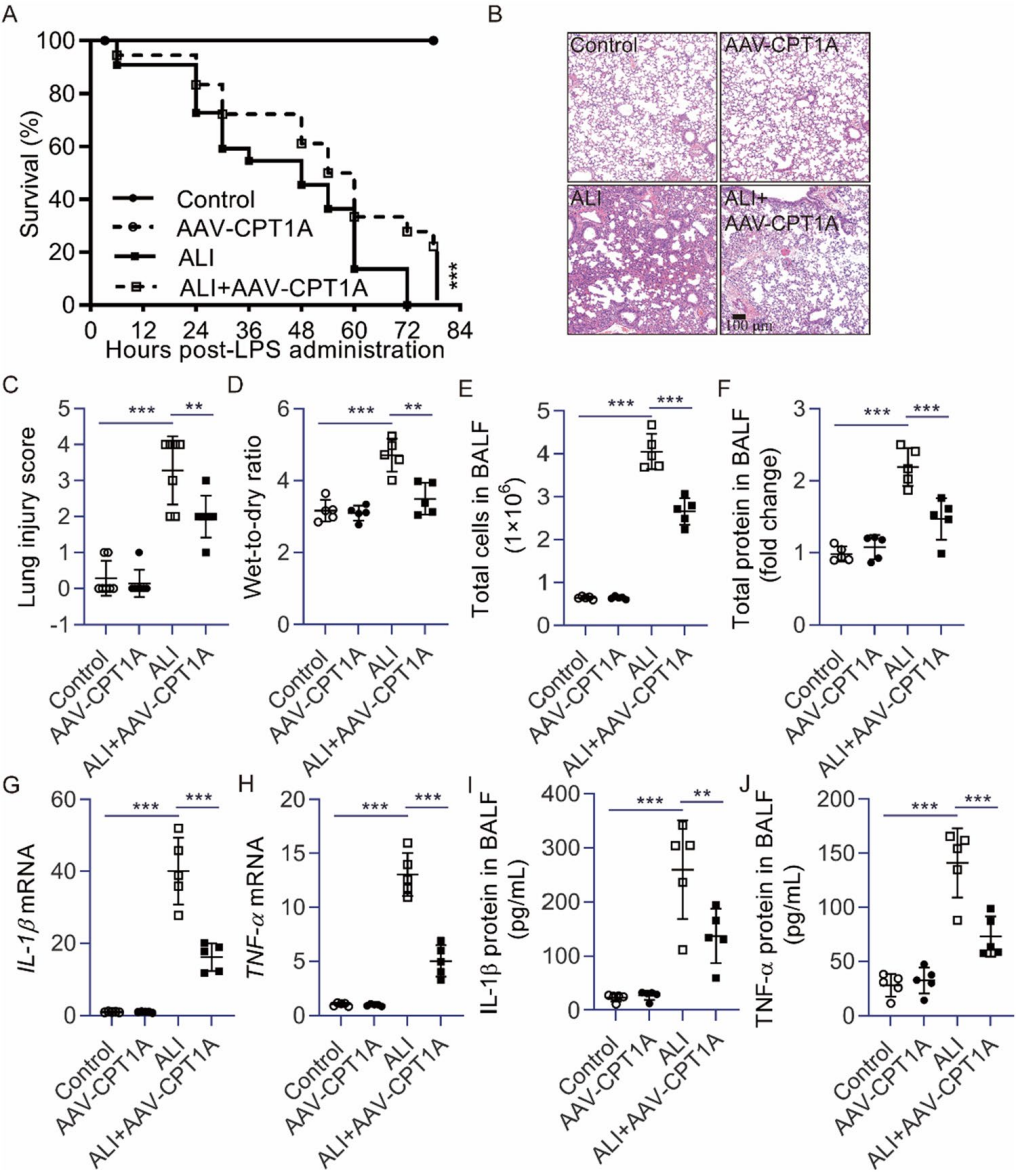
CPT1A overexpression alleviated lung injury and suppressed the inflammatory response in the lungs of ALI mice induced by LPS. (**A**) The survival rate of mice subjected to LPS. (**B**) The lung histopathological change by H&E staining. Scale bar: 100 μm. (**C**) Lung inflammation score. (**D**) The lung wet-to-dry ratio was determined. (**E**, **F**) BALF was collected to measure the number of cells or the amount of protein. (**G**, **H**) *Il-1β* and *Tnf-α* mRNA in lung tissue were determined. (**I**, **J**) Levels of cytokines (IL-1β and TNF-α) secretion in BALF were determined. *n* = 5, **P < 0.01 and ***P < 0.001.

It has been reported that CPT1A is remarkably reduced in lung tissue and AECs of ALI mice induced by lipopolysaccharide (LPS)^2^. To explore the effects of CPT1A in ALI induced by LPS, we injected CPT1A overexpression adeno-associated virus 9 (AAV-CPT1A) into mice with LPS-induced lung injury. The survival rate was significantly increased in the LPS + AAV-CPT1A group, compared with the ALI group in Fig. 1A. LPS induced evidently pathologic changes by increasing the infiltration of inflammatory cells and the lung injury score. However, LPS-induced severe histopathological changes were obviously weakened by the treatment of AAV-CPT1A (Fig. 1B,C). Additionally, AAV-CPT1A remarkably decreased the severity of edema formation, which was evaluated by the wet-to-dry ratio of the lungs in ALI mice (Fig. 1D). Decreased inflammatory cells and protein levels were observed in the bronchoalveolar lavage fluid (BALF) of ALI mice treated with AAV-CPT1A compared with ALI mice (Fig. 1E,F). Moreover, AAV-CPT1A not only markedly inhibited the expression of *Il-1β* and *Tnf-α* mRNA but also effectively reduced the releases of IL-1β and TNF-α (Fig. 1G–J). These observations support the notion that down-regulation of CPT1A mediates the development of ALI in vivo.

### CPT1A overexpression decreased cell apoptosis in the lungs of ALI mice

**Figure 2:**
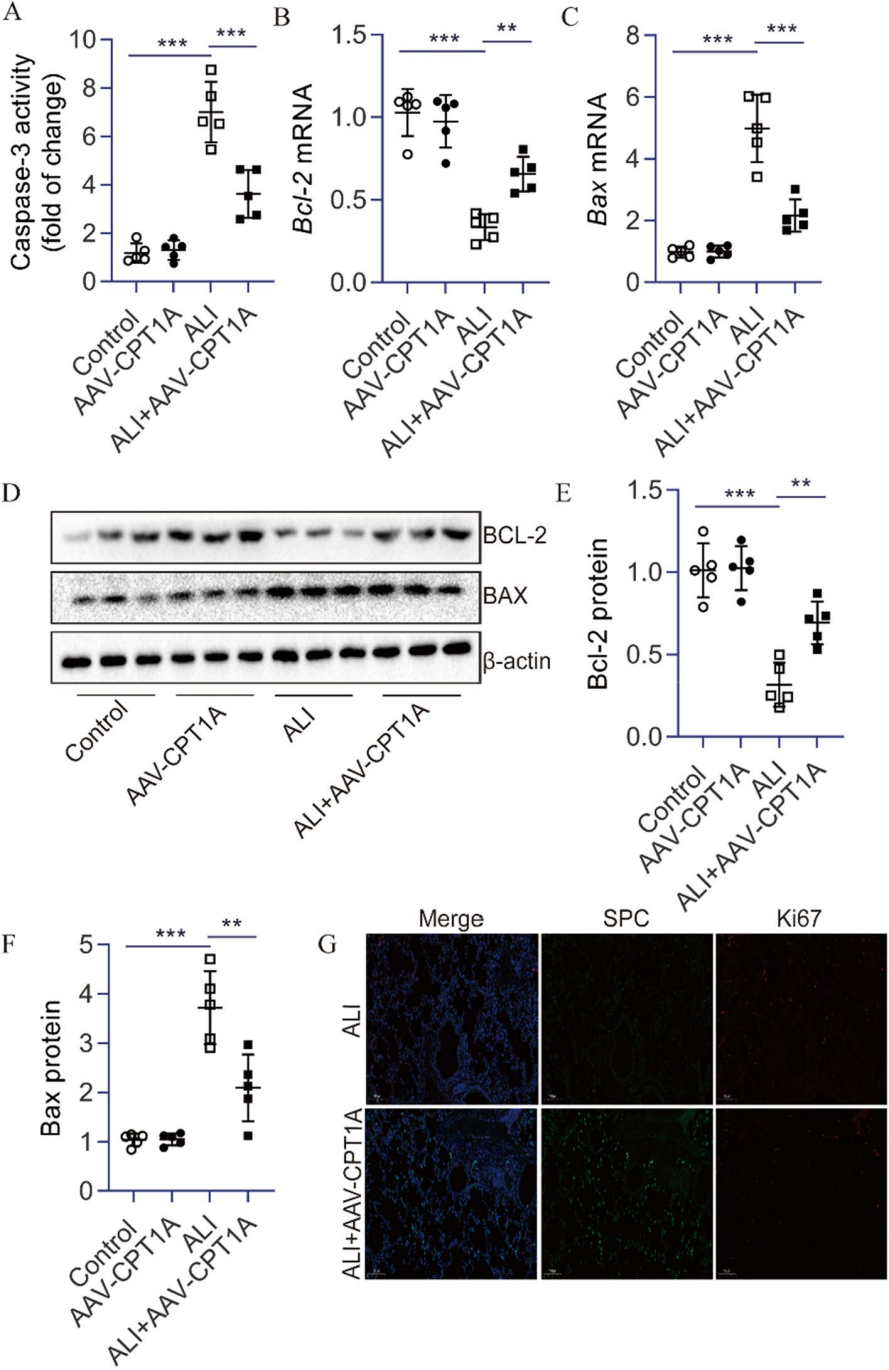
CPT1A overexpression decreased cell apoptosis in the lungs of ALI mice. (**A**) Caspase-3 activity in the lungs was determined. (**B**, **C**) *Bcl-2* and *Bax* mRNA in the lungs were determined. (**D**–**F**) BCL-2 and BAX proteins in the lungs were determined. (**G**) Immunofluorescence staining of lung tissue. SPC (Green) and Ki67 (Red). *n* = 5. **P < 0.01 and ***P < 0.001.

Next, we examined whether AAV-CPT1A pretreatment could reduce LPS-triggered cell apoptosis in lung tissue. Indeed, we found a more significant caspase-3 activity in ALI mice's lungs than in normal subjects (Fig. 2A). A decrease in BCL2 expression and an increase in BAX expression were exhibited in the lungs of ALI mice (Fig. 2B–F). In contrast, ALI mice treated with AAV-CPT1A showed decreased caspase-3 activity and BAX expression. AAV-CPT1A also reduced the decrease of BCL2 expression in the lungs of ALI mice (Fig. 2A–F). Furthermore, we found that overexpression of CPT1A failed to increase the Ki67 in AEC II of ALI mice (Fig. 2G). Collectively, these data indicate that CPT1A plays an anti-apoptotic role in the lungs of ALI mice induced by LPS.

### Blocking CPT1A with etomoxir augmented inflammatory responses and lung injury in ALI mice

**Figure 3:**
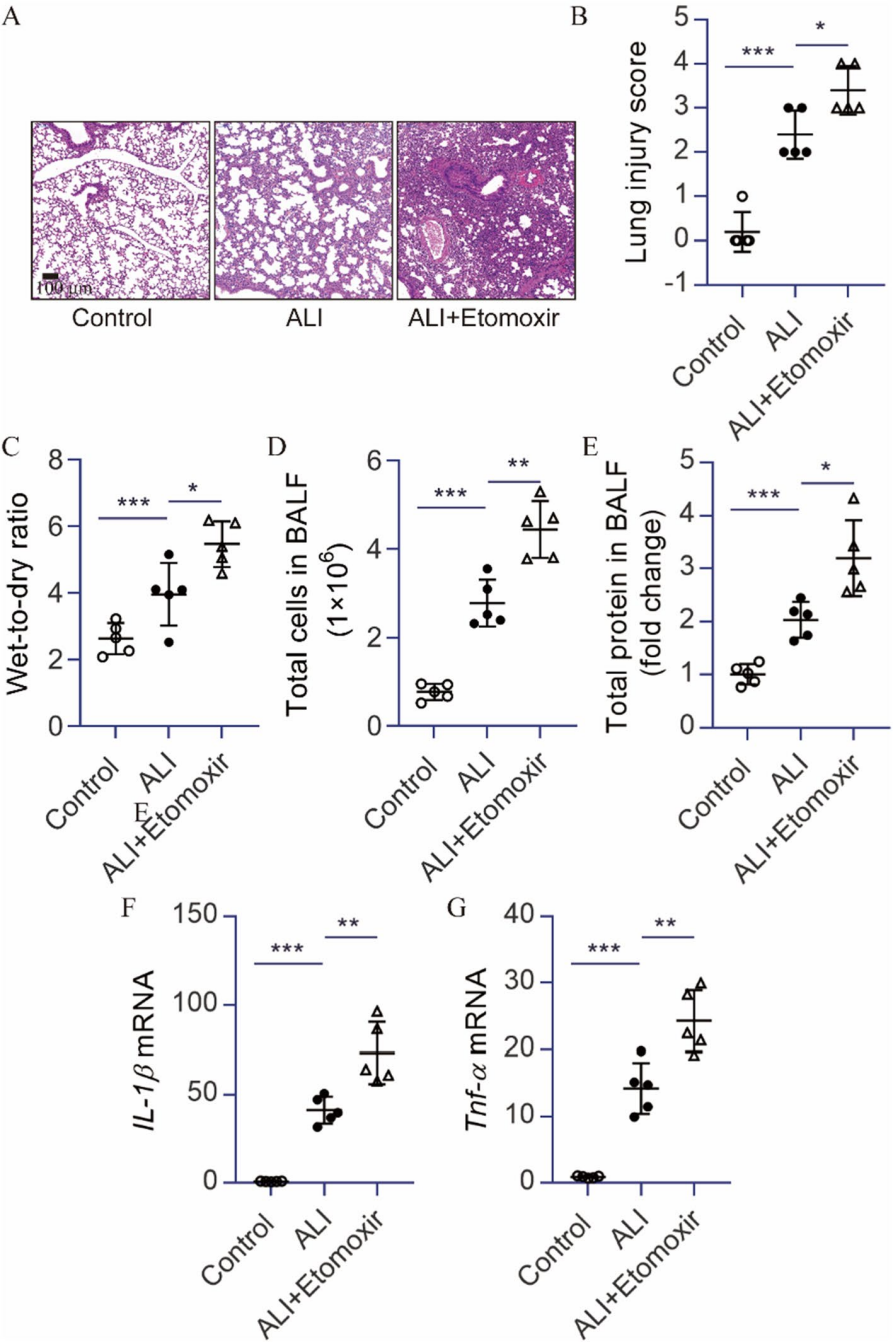
Blocking CPT1A with etomoxir augmented inflammatory responses and lung injury in ALI mice*.* (**A**) The lung histopathological change by H&E staining. Scale bar: 100 μm. (**B**) Lung inflammation score. (**C**) The lung wet-to-dry ratio was determined. (**D**, **E**) BALF was collected to measure the number of cells or the amount of protein. (**F**, **G**) *Il-1β* and *Tnf-α* mRNA in the lungs were determined. *n* = 5, *P < 0.05, **P < 0.01, and ***P < 0.001.

Based on these results in vivo, we further investigated whether blocking CPT1A with its small-molecule inhibitor etomoxir could augment lung injury induced by LPS. Mice treated with etomoxir were susceptible to LPS-induced ALI. There was a significant increase in the histopathological changes and wet-to-dry ratio of lung tissue in LPS-induced mice (Fig. 3A–C). Increased inflammatory cells and protein levels were observed in the BALF of mice treated with etomoxir subjected to LPS compared with ALI mice (Fig. 3D,E). Furthermore, there were significantly elevated mRNA levels of *Il-1β* and* Tnf-α* in the lungs of mice treated with etomoxir subjected to LPS (Fig. 3F,G). Taken together, these results indicate that blocking CPT1A is more susceptible to LPS-induced inflammatory response and lung injury in mice.

### Overexpression of CPT1A protected MLE12 cells against LPS-induced apoptosis in vitro

**Figure 4:**
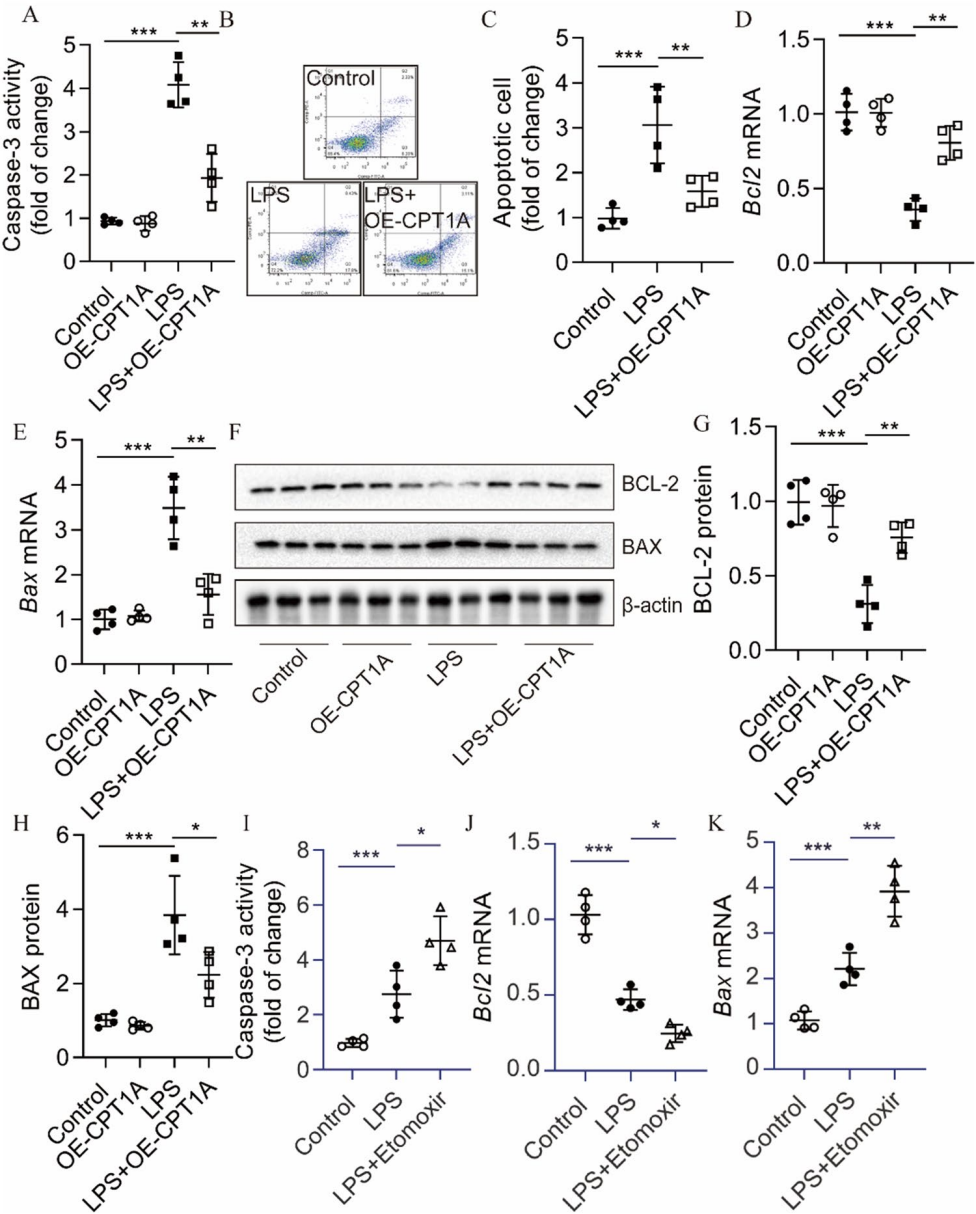
Effects of CPT1A on LPS-induced apoptosis in MLE12 cells in vitro. (**A**) Caspase-3 activity in MLE12 cells treated with Lenti-CPT1A was determined. (**B**, **C**) The apoptosis rates of MLE12 cells treated with Lenti-CPT1A were determined by flow cytometry. (**D**, **E**) *Bcl-2* and *Bax* mRNA in MLE12 cells treated with Lenti-CPT1A were determined. (**F**–**H**) BCL-2 and BAX protein in MLE12 cells treated with Lenti-CPT1A were determined. (**I**) Caspase-3 activity in MLE12 cells treated with etomoxir (10 μM) was determined. (J-K) *Bcl-2* and *Bax* mRNA in MLE12 cells treated with etomoxir (10 μM) were determined. *n* = 4, *P < 0.05, **P < 0.01, and ***P < 0.001.

Then, we wondered whether CPT1A exerted a protective effect against AEC apoptosis in vitro. We found that treating MLE12 cells with LPS alone significantly increased caspase-3 activity and apoptotic ratio, whereas overexpression of CPT1A via lentivirus (OE-CPT1A) considerably inhibited the LPS-induced increase in caspase-3 activity and apoptotic ratio (Fig. 4A–C). In addition, OE-CPT1A pretreatment could dramatically revise the LPS-induced upregulation of BAX expression and the downregulation of BCL-2 expression in MLE12 cells (Fig. 4D–H). As illustrated in Fig. 4I–K, compared with the LPS group, pretreated with etomoxir dramatically increased caspase-3 activity and augmented the change of BCL2 and BAX expression. These data indicate that the protective effects of CPT1A overexpression on LPS-induced ALI may be associated with suppressing the apoptosis in epithelial cells.

### CPT1A overexpression mitigated mitochondrial dysfunction and restored FAO in lung tissue of ALI mice and LPS-treated MLE12 cells

**Figure 5:**
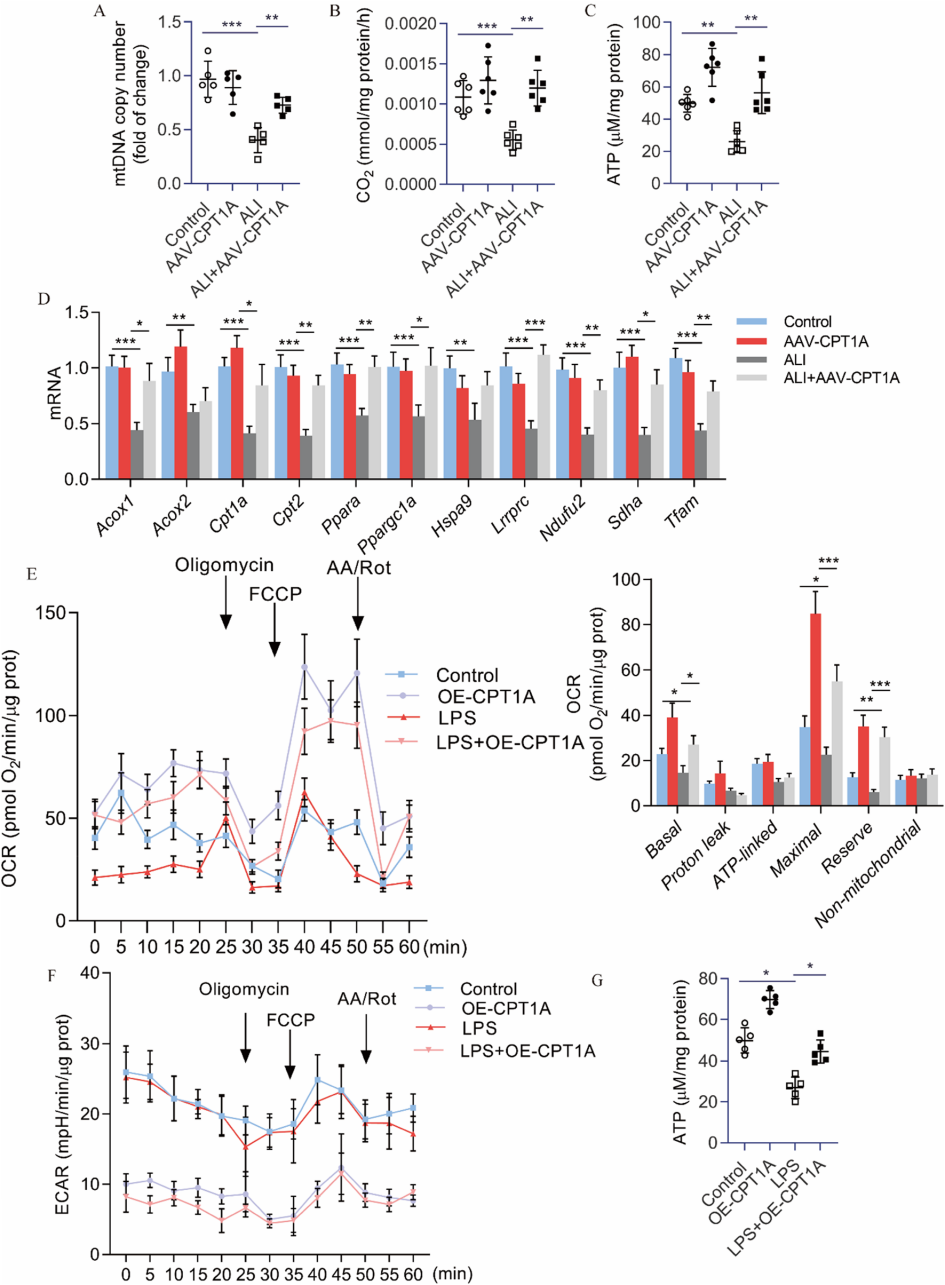
CPT1A overexpression mitigated mitochondrial dysfunction and restored FAO in lung tissue of ALI mice and LPS-treated MLE12 cells. (**A**) Mitochondrial DNA copy number (mtDNA) was determined in the lungs. (**B**) Radiolabeled palmitate-derived CO_2_ was determined after incubating ^14^C-palmitate with lung tissue after doxycycline induction. (**C**) ATP levels in total lung tissue. (**D**) Peroxisomal/mitochondrial function-associated genes were determined in the lungs after doxycycline induction. (**E**) OCR of MLE12 cells was measured with a Seahorse XF24 Extracellular Flux Analyzer. Bar graphs show the rates of OCR associated with basal, proton-leak, ATP-linked, maximum, reserve capacity, and nonmitochondrial respiratory statuses. (**F**) Extracellular acidification rate (ECAR) of MLE12 cells. (**G**) ATP levels of MLE12 cells.* n* = 6, *P < 0.05, **P < 0.01, and ***P < 0.001.

CPT1A overexpression decreased the reduction in mtDNA copy number in the lungs of ALI mice (Fig. 5A). Defective FAO was observed in the lungs of ALI mice, while CPT1A overexpression alleviated this impairment (Fig. 5B). Furthermore, CPT1A overexpression prevented the drop in ATP content in whole lung tissue (Fig. 5C). As expected, LPS significantly reduced the expression of genes involved in peroxisomal/mitochondrial function-related genes, including *Acox1*, *Cpt2*, *Ppara*, *Ppargc1a*, *Lrpprc*, *Ndufu2*, *Sdha*, and *Tfam* (Fig. 5D). CPT1A overexpression recovered the decreased expression of these genes (Fig. 5D). Next, we investigated the metabolic functional consequences of CPT1A overexpression in the MLE12 cells in vitro. We found that MLE12 cells with CPT1A overexpression exhibited reduced LPS-induced FAO inhibition (Fig. 5E) and suppression of glycolysis, as reflected by ECAR levels (Fig. 5F). The decrease in ATP levels related to treatment with LPS was also recovered under CPT1A overexpression (Fig. 5G). Collectively, these data indicate that CPT1A overexpression mitigates mitochondrial dysfunction and restores FAO in lung tissue of ALI mice and LPS-treated MLE12 cells.

## Discussion

In the current study, we found that overexpression of CPT1A alleviated LPS-induced pulmonary edema, inflammation, and inflammation cell infiltration. The elevation of lung wet-to-dry ratio and protein level in BALF were markedly decreased by overexpression of CPT1A. Overexpression of CPT1A inhibited the expressions of pro-inflammatory cytokines, such as IL-1β and TNF-α, in the lungs of ALI mice. Furthermore, the blockade of CPT1A aggravated ALI induced by LPS in vitro and in vivo. Besides, CPT1A played an anti-apoptotic role in the lungs and MLE12 cells induced by LPS. Taken together, these investigations imply that CPT1A might be a potential target of ALI.

Studies in ALI have focused on the role of epithelial cells as a critical feature in disease pathogenesis^14,26^. It has been reported that epithelial cell apoptosis is required for the development of ALI^14,15^. Epithelial cells exhibit two apoptosis pathways, intrinsic and extrinsic, during ALI^27–29^. Protecting AEC II cells from apoptosis could alleviate the ALI in mice^16,18^. CPT1A is the rate-limiting enzyme for FAO, which resides in the outer mitochondrial membrane. CPT1A initiates FAO by converting long-chain acyl-CoA to long-chain acyl-carnitines. A previous study has shown that FAO is strikingly impaired in AECs of mice with LPS, and CPT1A is decreased in LPS-treated MLE12 cells^2^. Inhibition of FAO increases the de novo synthesis of ceramide, leading to apoptosis^30^, whereas augmenting FAO protects against palmitate-induced apoptosis^31^. Further, enhancing FAO protects against hyperoxia-induced apoptosis in mouse lungs^32^. Overexpression of CPT1A alleviated lung dysfunction and reduced inflammatory response and apoptosis of lung tissue in COPD mice^33^. Consistent with these studies, our results showed that CPT1A overexpression could reduce LPS-induced apoptosis in MLE12 cells, while etomoxir increased apoptosis-related gene expression induced by LPS in MLE12 cells. In addition, *Cpt1a* deficiency can inhibit endothelial cell proliferation and angiogenesis^34^. Another study found that CPT1A inhibits the proliferation and migration of lung cancer cells^35^. In view of these findings, the role of CPT1A in proliferation is controversial. Here, we found that overexpression of CPT1A failed to increase the Ki67 induced by LPS in type II alveolar epithelial cells of ALI mice. Thus, the protective effect of CPT1A is partially associated with its anti-apoptotic pathways.

The initiation of apoptosis is closely associated with mitochondrial dysfunction^36^. The Bcl-2 family of proteins, including anti-apoptotic proteins (Bcl-2 and Bcl-xl) and proapoptotic proteins (Bad, Bax, and Bid), play an essential role in programmed cell death^37,38^. Caspase-3 is referred to as the executor of cell apoptosis^39^. A previous study has demonstrated that CPT1A mediates apoptosis resistance in monocyte-derived macrophages by directly binding to Bcl-2^23^. And *Cpt1a* deficiency promotes fibroblast apoptosis in a fibrosis model^23^. In our study, we also observed the upregulation of BAX and the downregulation of BCL-2 in MLE12 cells treated with LPS and lung tissue of ALI mice. Our study further showed that CPT1A overexpression decreased cell apoptosis in ALI mice lung tissue and MLE12 cells treated with LPS while blocking CPT1A increased apoptosis in vitro. We concluded that CPT1A provokes apoptosis resistance in epithelial cells of ALI mice.

Mitochondrial abnormalities and dysfunction are features in the pathogenesis of ALI^40,41^. Compromising mitochondrial homeostasis promotes lung damage, reflected in increased oxidative stress and apoptosis, which contribute to the deterioration of lung function^1,42^. It has been reported that FAO is significantly impaired in AECs of ALI lungs, and the defective FAO leads to apoptosis of AECs^2^. In our study, we showed that overexpression of CPT1A enhanced OCR and increased ATP production in conditions of lung injury. On the contrary, CPT1A overexpression exhibited lower ECAR. This appeared to occur at the expense of glycolysis, which was also confirmed in kidney fibrosis^2^.

In conclusion, our findings demonstrated that overexpression of CPT1A effectively protected against lung injury in ALI mice by suppressing epithelial cell apoptosis. This study indicates that CPT1A is a promising therapeutic target for LPS-induced ALI.

## Methods

### Ethics statement

The animal studies were approved by the Ethics Committee of animal experiments at Central South University, and all the processes are in strict accordance with the National Institutes of Health (NIH) Guide for the Care and Use of Animals in laboratory experiments. All methods were reported in accordance with ARRIVE guidelines. During all procedures, pain was minimized using the anesthetic sodium pentobarbital. Mice were euthanized by injection of sodium pentobarbital.

### Animal and experimental model

ALI model of mice was induced as previously described^43^ via LPS (*E. coli O111:B4*; 5 mg/kg; Sigma-Aldrich, St. Louis, MO, USA) intratracheal injection (*i.t.*). Twelve hours after LPS treatment, the mice were sacrificed for further detection. To explore the role of CPT1A in ALI mice, mice were randomly divided into 4 groups: control, ALI, AAV-CPT1A, and ALI + AAV-CPT1A groups. Overexpression of CPT1A was administered by 1.5 × 10^12^ genome copies (gc)/kg of AAV9 via tail-vein injection. The mice in the control group were treated with saline and the control AAV9. The ALI mice were treated with LPS and the control AAV9. To explore the role of CPT1A blocking on ALI mice, mice were randomly divided into 3 groups: control, ALI, and ALI + etomoxir groups. Mice in the ALI + etomoxir group received etomoxir (20 mg/kg; MedChemExpress, USA) via intraperitoneal injection 1 h before saline or LPS administration. For the survival study, mice were treated with LPS at a lethal dose (25 mg/kg, *i.t.*). Mice in the control group received saline only. The survival rate was monitored every 6 h.

### Histological analysis

Lung tissue samples from the left lungs of mice were fixed in 4% paraformaldehyde, dehydrated in a series of graded ethanol, embedded in paraffin wax, and cut into 5-μm-thick sections. The sections were stained with Hematoxylin–Eosin (HE). Lung injury scores were evaluated by three researchers. Each slide was scored for five randomly selected fields at 400 × magnification.

### Lung wet-to-dry ratio

Whole-lung tissue was collected, and surface blood was removed. Then, the weights of the samples were recorded as the wet weight. Lung tissue was dried for 72 h at 65 °C and recorded as the dry weight.

### Collection and analysis of BALF

The BALF was collected by flushing the lungs three times with 1 mL of cold sterile physiological saline (0.9% NaCl) via the tracheal cannula. After removing the erythrocytes in the BALF by lysis buffer, the total cell number was counted. BALF protein concentrations were determined by the bicinchoninic acid (BCA) protein assay kit (Beyotime Biotechnology, China).

### Immunofluorescence

Lung tissue was fixed overnight in 4% paraformaldehyde and then embedded in paraffin. Serial 4-μm sections were mounted on slides. Localization of SPC and Ki67 in lung tissue was performed by a double-labeled immunofluorescence method. Sections were incubated overnight at 4 °C with a cocktail of two antibodies: rabbit anti-SPC antibody (1:50, Abcam, USA) and rat anti-Ki67 antibody (1:20, Abcam, USA). Then, sections were incubated with FITC and Cy3-conjugated secondary antibodies after washing in PBS. Sections were viewed with a fluorescence microscope (Thermo, USA).

### Cell culture and treatments

Mouse lung epithelial cell line MLE12 cells were obtained from American Type Culture Collection (ATCC). MLE12 cells were cultured in DMEM-F12 medium (HyClone, USA) supplemented with 10% fetal bovine serum (FBS, Gibco, USA). To achieve the overexpression of CPT1A, we used an overexpression lentivirus vector. While a CPT1A inhibitor etomoxir (10 μM)^44^ was used to block the activity of CPT1A.

### Enzyme-linked immunosorbent assay (ELISA)

Levels of IL-1β and TNF-α in the BALF were evaluated using ELISA in accordance with the manufacturer’s instructions (R&D Systems, USA).

### Quantitative RT-PCR (qPCR)

Total RNA was extracted from tissues and cells using the Trizol reagent, and then cDNA was generated using a reverse transcription kit (Takara, Japan). The qPCR was performed using SYBR Green PCR Master Mix (Cwbiotech, Beijing, China) in the CFX96 Touch Real-Time PCR Detection System (Bio-Rad, USA). The primer sequences are listed as follows: *Tnf-α*, cttctcattcctgcttgtg, acttggtggtttgctacg; *Il-1β*, cctgggctgtcctgatgagag, tccacgggaaagacacaggta; *Bcl-2*, aagctgtccacaggagggcta, cacagagctcatgtgtcccac; *Bax*, tgcagaaggatgattgctgac, cacgccatcctctccagat; *Gapdh*, caatgtgtccgtcgtggatct, gtcctcagtgtagcccaagatg. The expression levels of genes were normalized to *Gapdh* with the 2^-ΔΔCt^ method.

### The caspase-3 activity detection

The lung tissue and cells were lysed in a cell lysis buffer. The reaction buffer and DEVD-ρNA substrate (caspase-3) were supplemented into the lysis buffer. After cultured at 37 °C for 2 h, the absorbance at 405 nm was assessed via a microplate reader (Meigu, Shanghai, China).

### Western blot analysis

Total proteins extracted from harvested tissues and cells were prepared, and the protein concentration was determined using the BCA protein assay kit (Beyotime Biotechnology, China). Subsequently, sodium dodecyl sulfate–polyacrylamide gel electrophoresis was performed, and 40 μg protein was loaded. The membranes were incubated with primary antibody (BCL2, 1:1000, CST; BAX, 1:1000, CST; β-actin,1:7500, Sigma-Aldrich) at 4 °C overnight. Afterward, the membranes were incubated with secondary antibodies for 2 h. The protein levels were detected with an enhanced chemiluminescence reagent, and the intensities were quantified using Image J gel analysis software (representative images for each western blot result presented in Supplementary material).

### Cell apoptosis detection

Cells were collected and labeled by binding buffer, including 5 μL of Annexin V-FITC and 5 μL of propidium iodide (Roche, USA) to each sample. After being mixed gently and incubated at room temperature in the dark for 15 min, cells were immediately analyzed on flow cytometry (Beckman Coulter MoFloTM XDP, USA).

### Mitochondrial DNA copy number determination

DNA was extracted from lung tissue using the DNeasy Blood and Tissue Kit (Qiagen, USA). The Mouse Mitochondrial DNA Copy Number Assay Kit (Detroit R&D) was used to determine mitochondrial abundance.

### ATP content detection

ATP levels in lung tissue were measured using Luminescent ATP Detection Assay Kit (Abcam, USA) according to the manufacturer’s instructions. Data were normalized for total protein content.

### OCR analysis

Oxygen consumption rate (OCR) and extracellular acidification rate (ECAR) were measured by XF-24 Extracellular Flux Analyzer (Seahorse Bioscience, USA).

### Statistical analysis

All the data, displayed as mean ± standard deviation, were analyzed applying SPSS 25.0. The significance of the difference was analyzed by ANOVA, followed by Tukey–Kramer’s post-hoc test. The results were considered statistically significant when *P* < 0.05.

### Supplementary Information

Supplementary Figures.

## Data Availability

The data supporting this study's findings are available from the corresponding author upon reasonable request.
