# Supplementary material for: CPT1A as a potential therapeutic target for lipopolysaccharide-induced acute lung injury in mice
Source: Sci Rep. 2024 Jan 18;14:1600. doi: 10.1038/s41598-024-52042-2 (PMC10796431; doi:10.1038/s41598-024-52042-2)

**Supplementary Information**

Figure 2D-bax


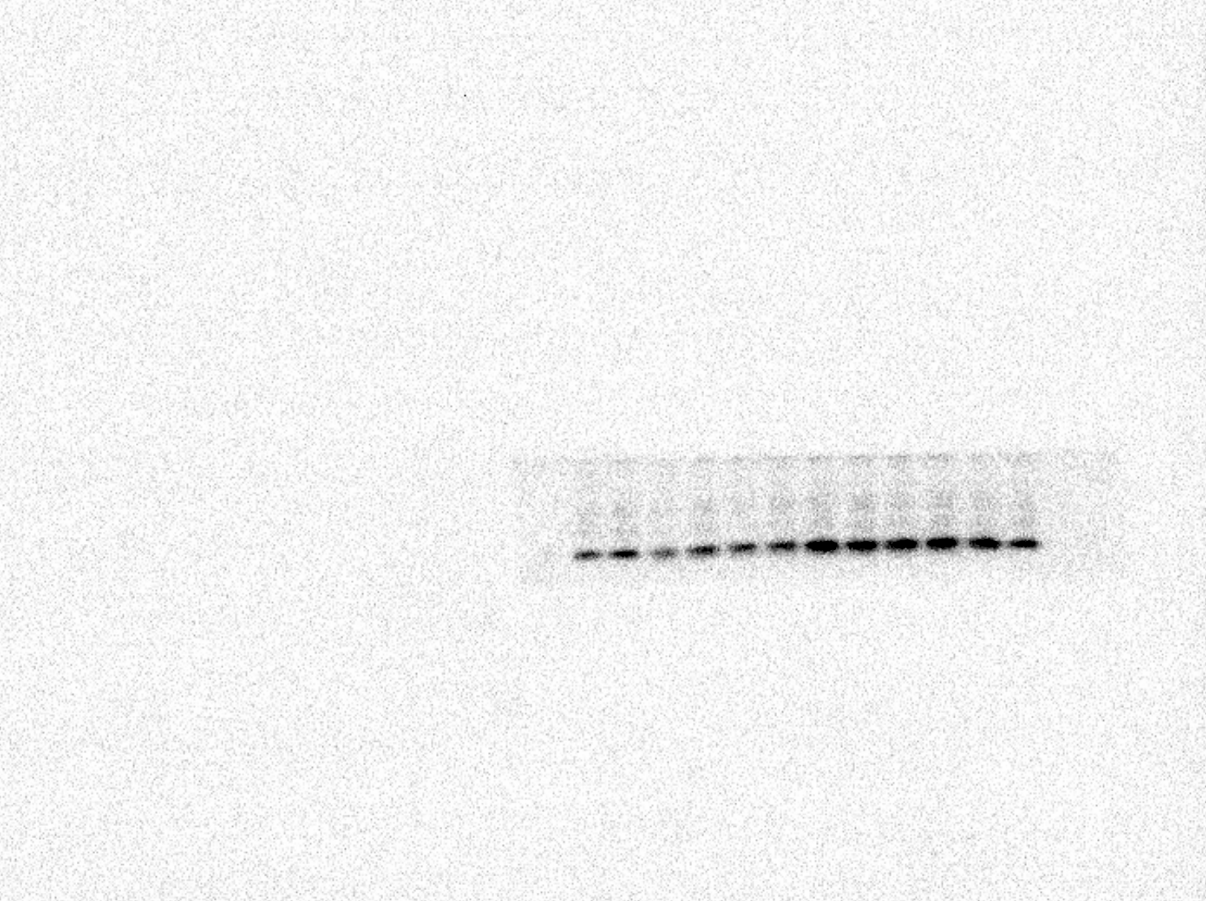


Figure 2D-bcl2


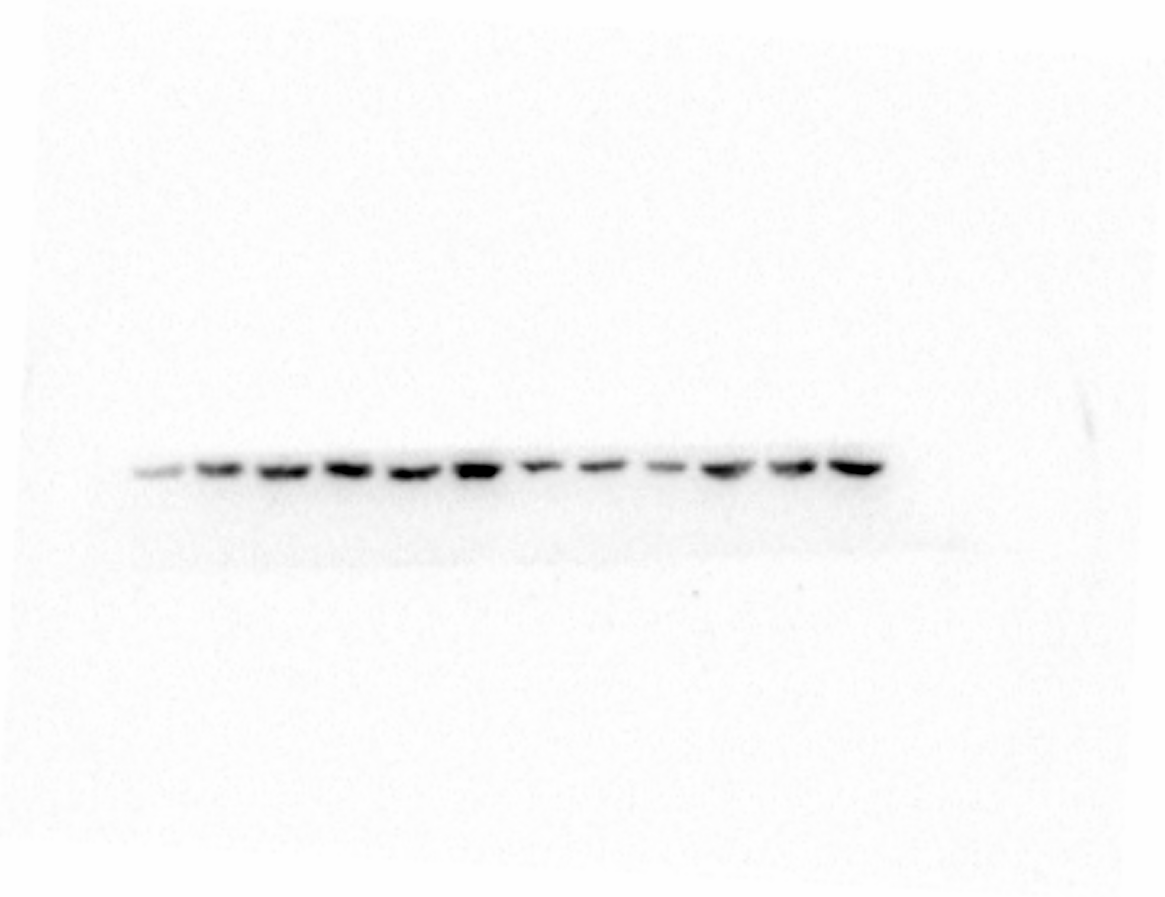


Figure 2D-β-actin


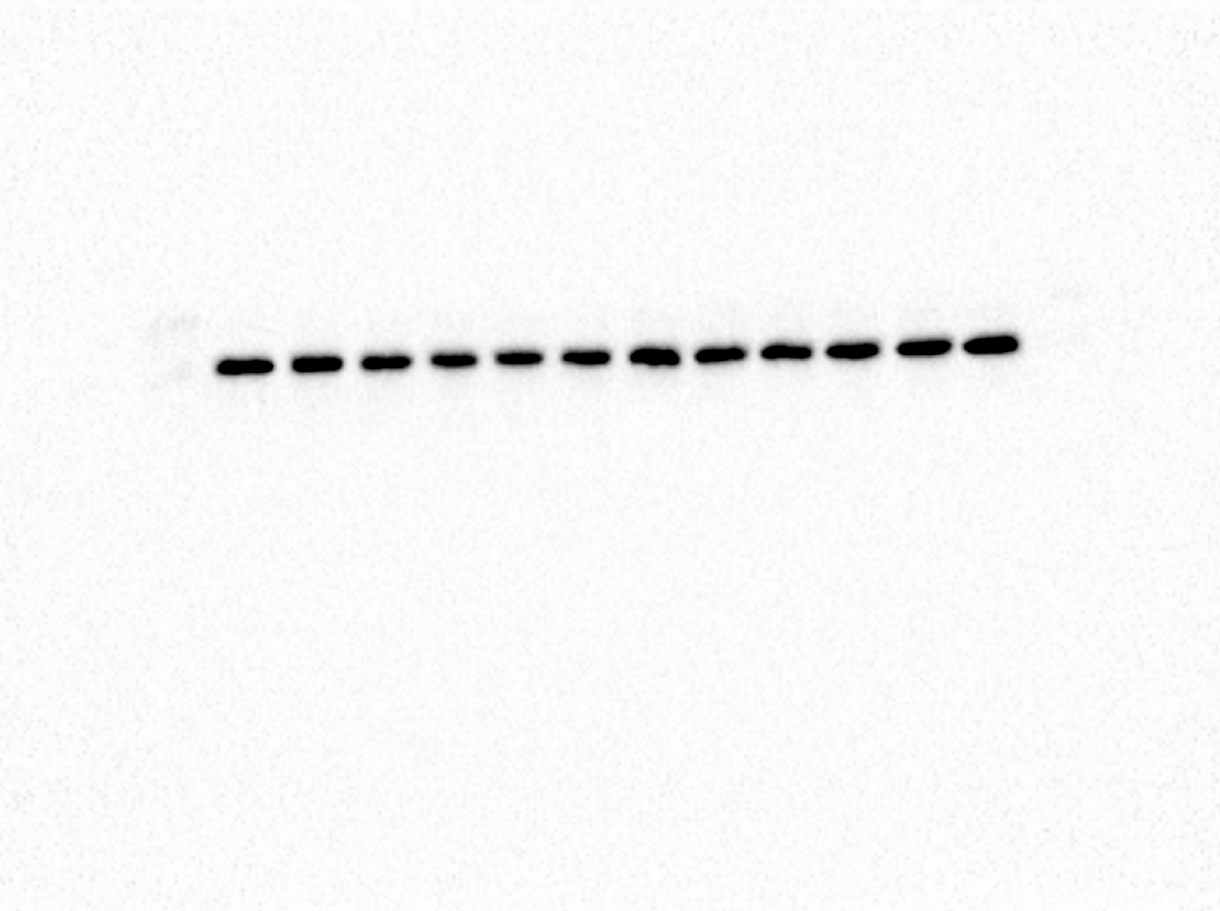


Figure 4F-bax


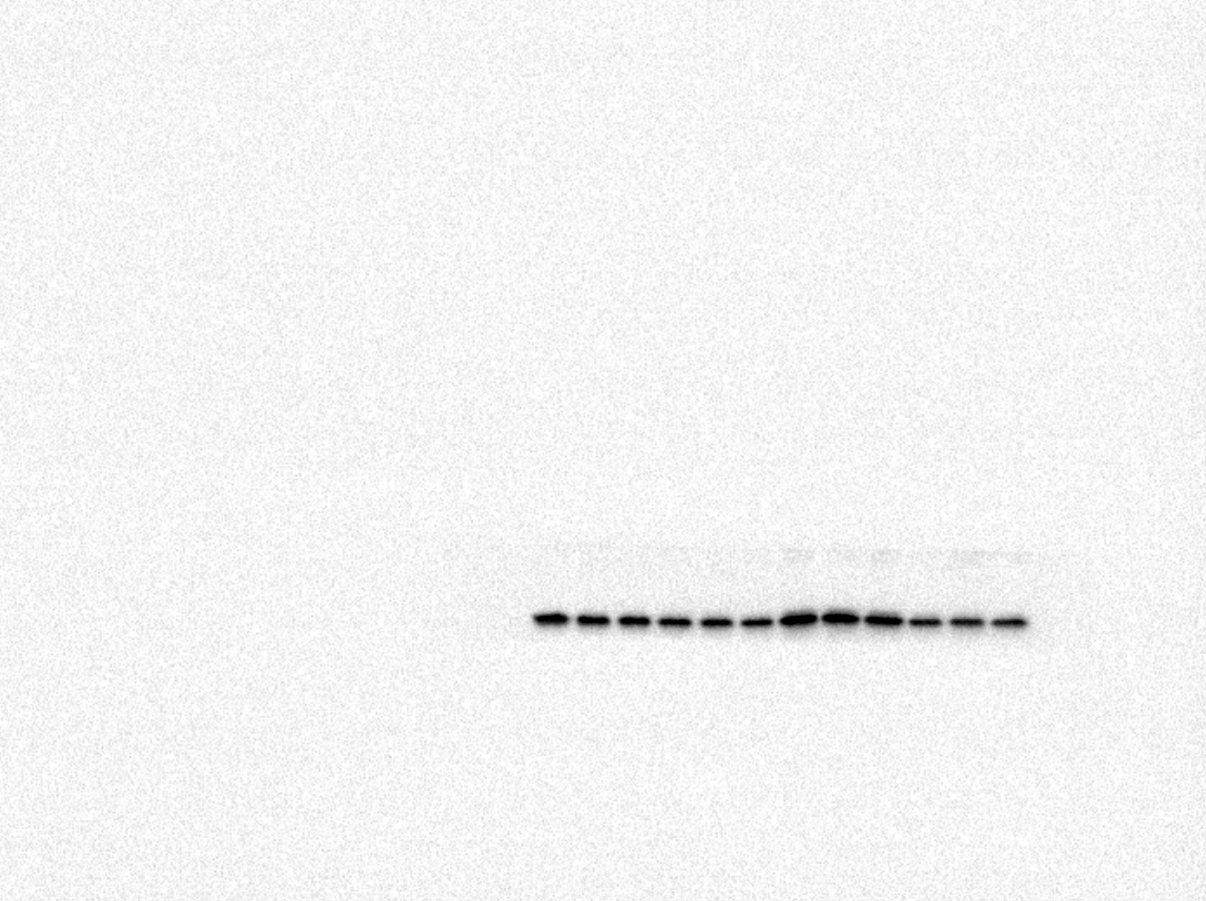


Figure 4F-bcl2


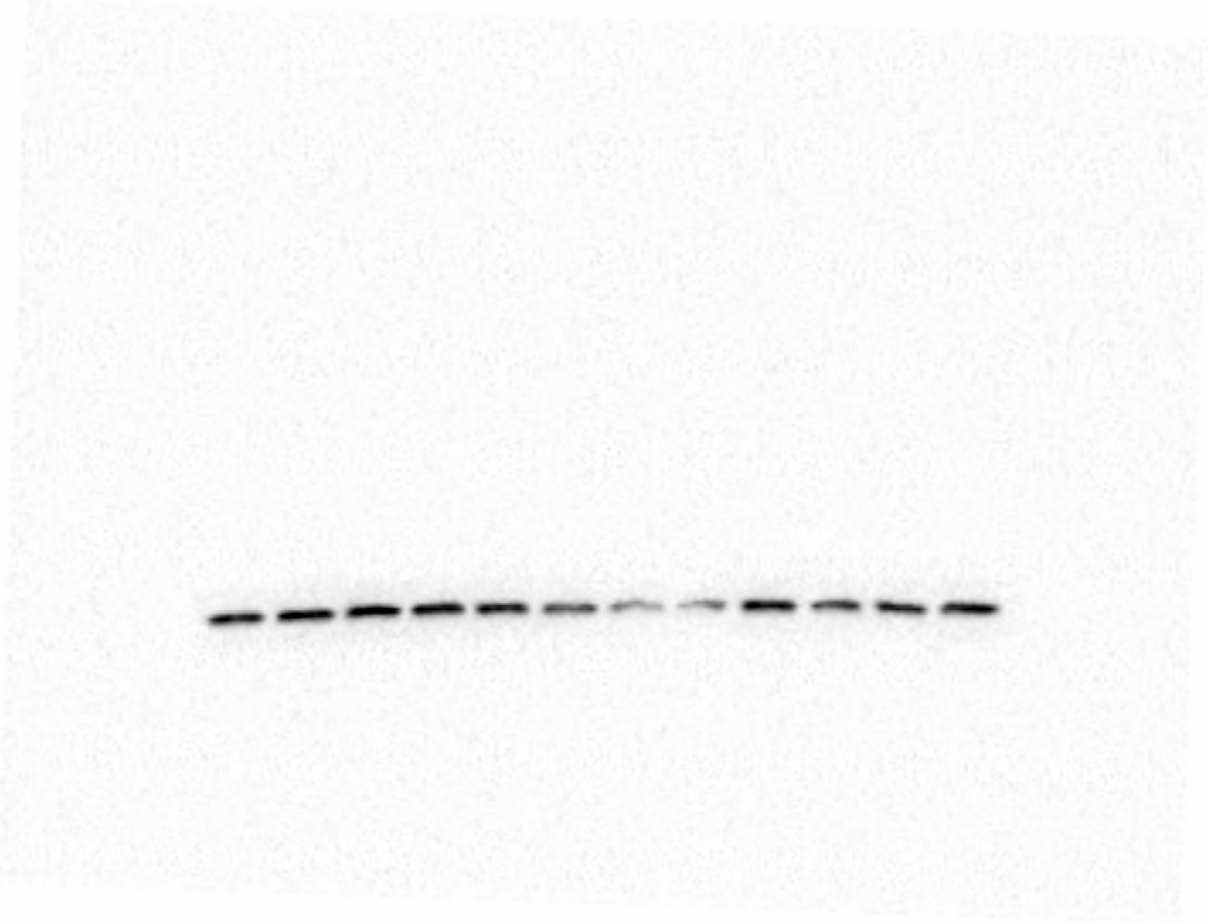


Figure 4F-β-actin


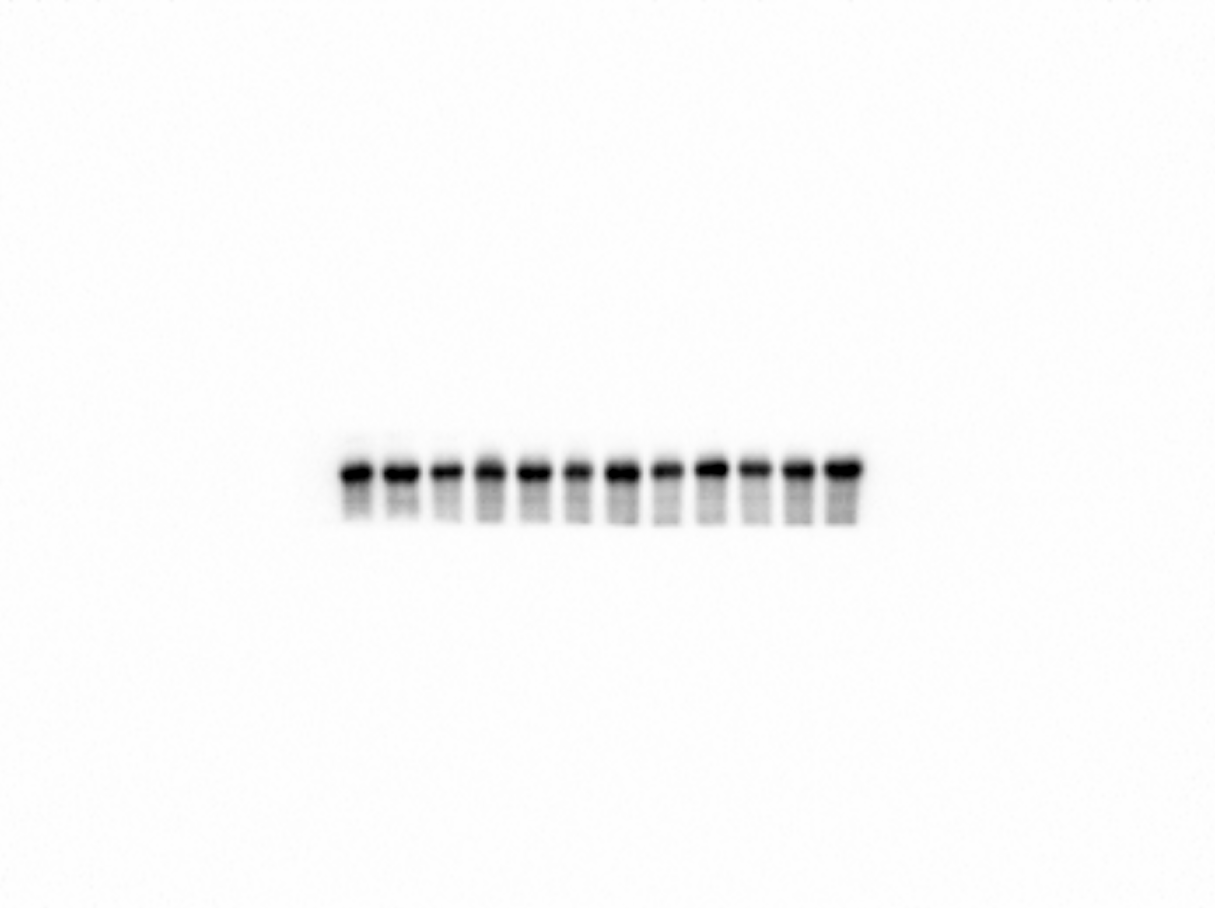

Supplement: Supplementary file 1 — Supplementary Figures. [file 41598_2024_52042_MOESM1_ESM.docx]
